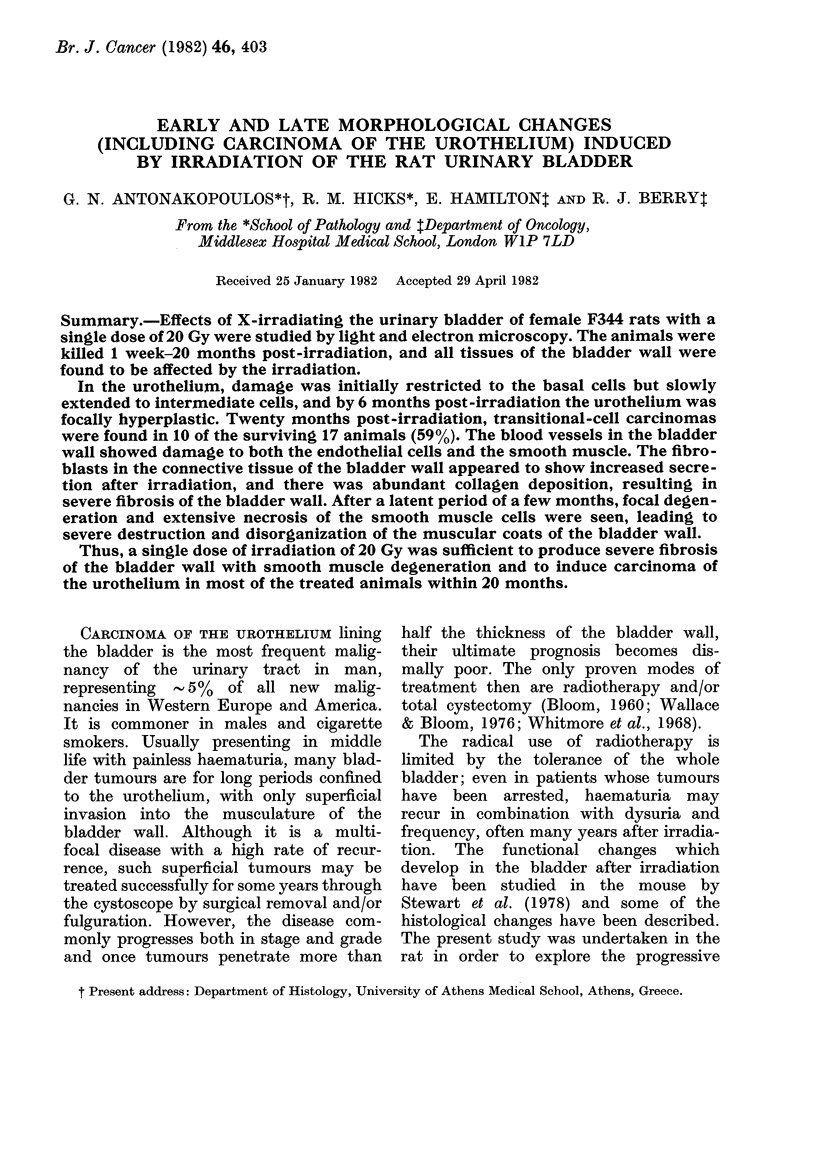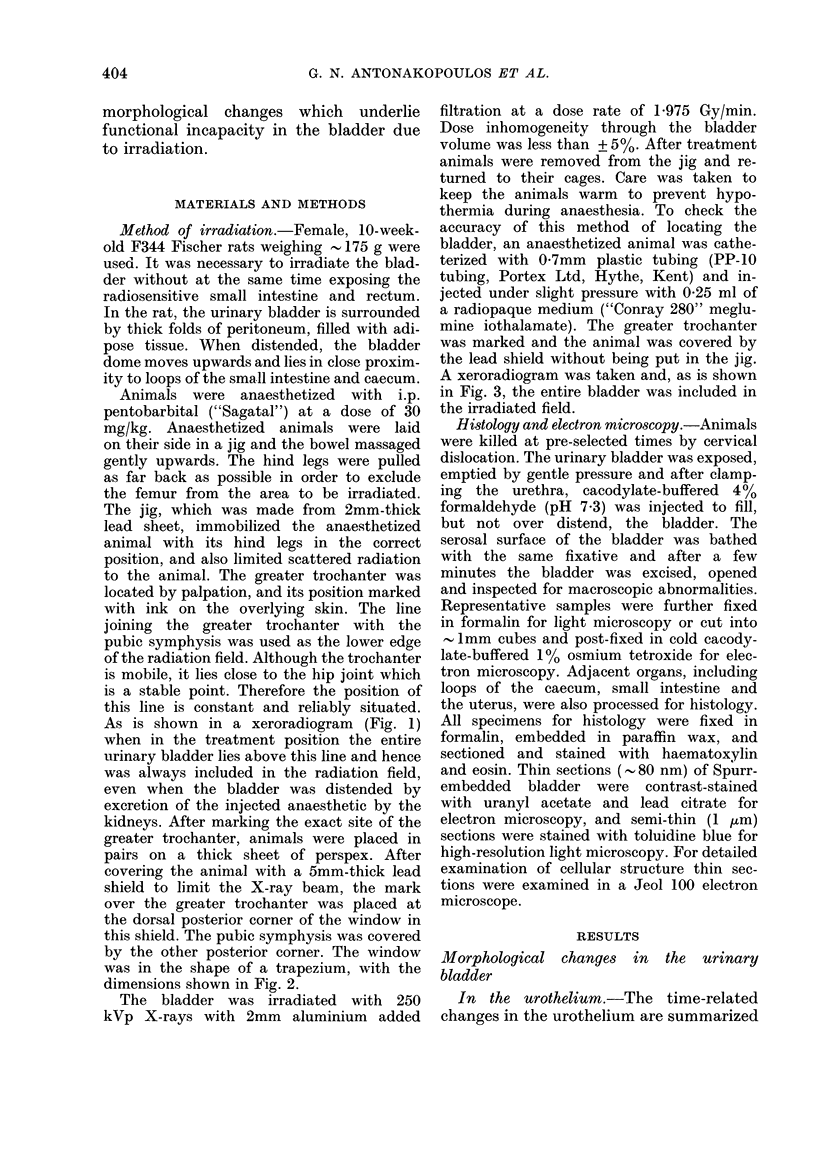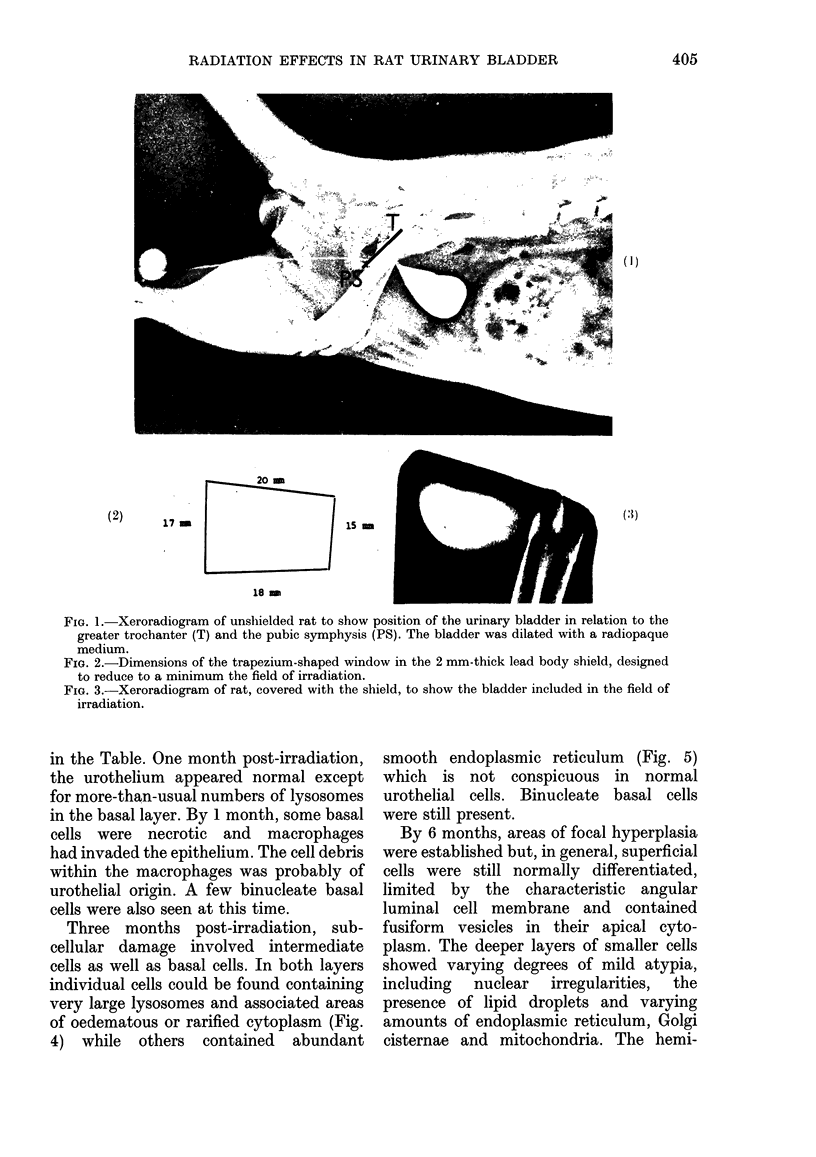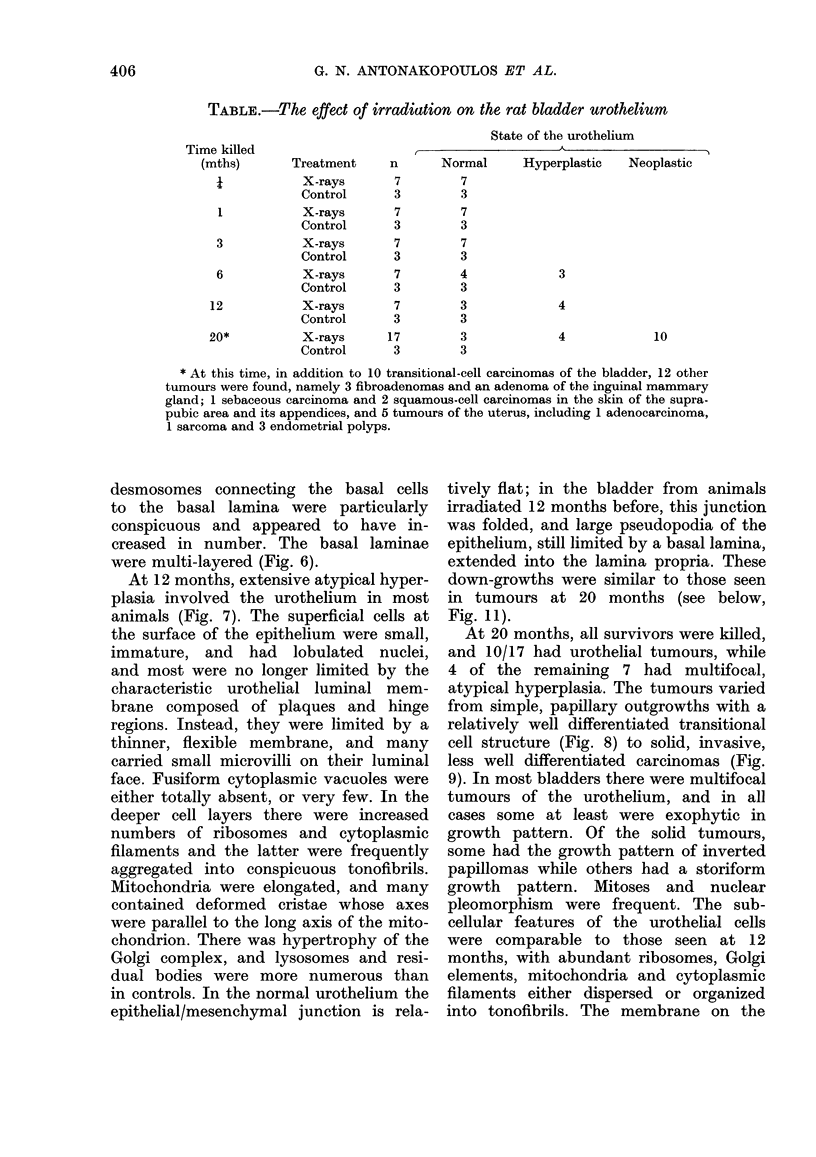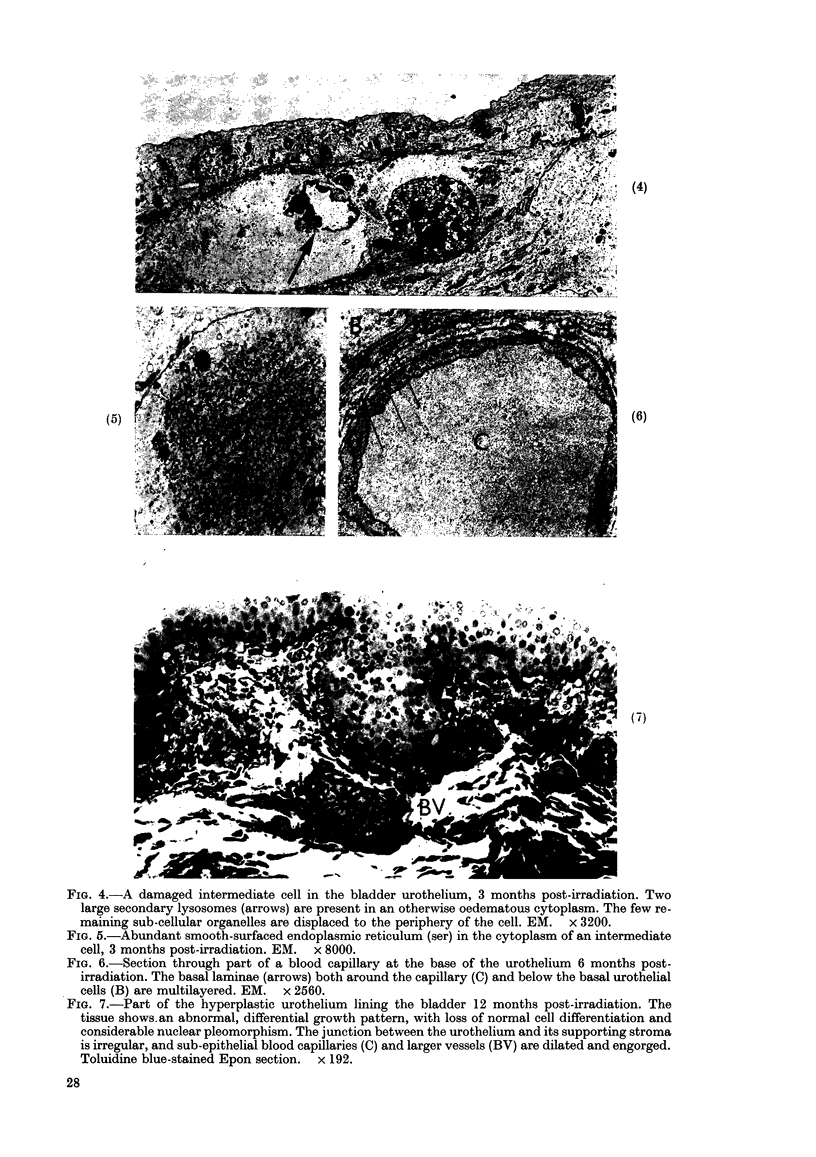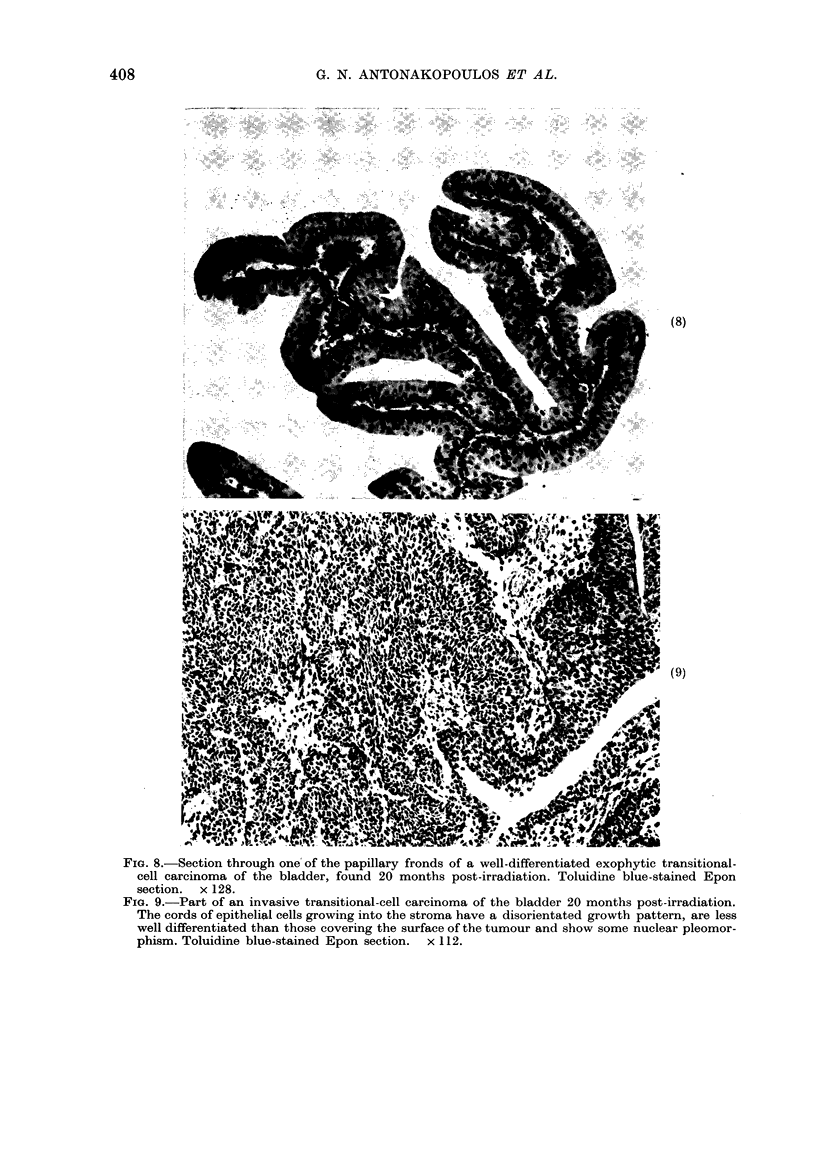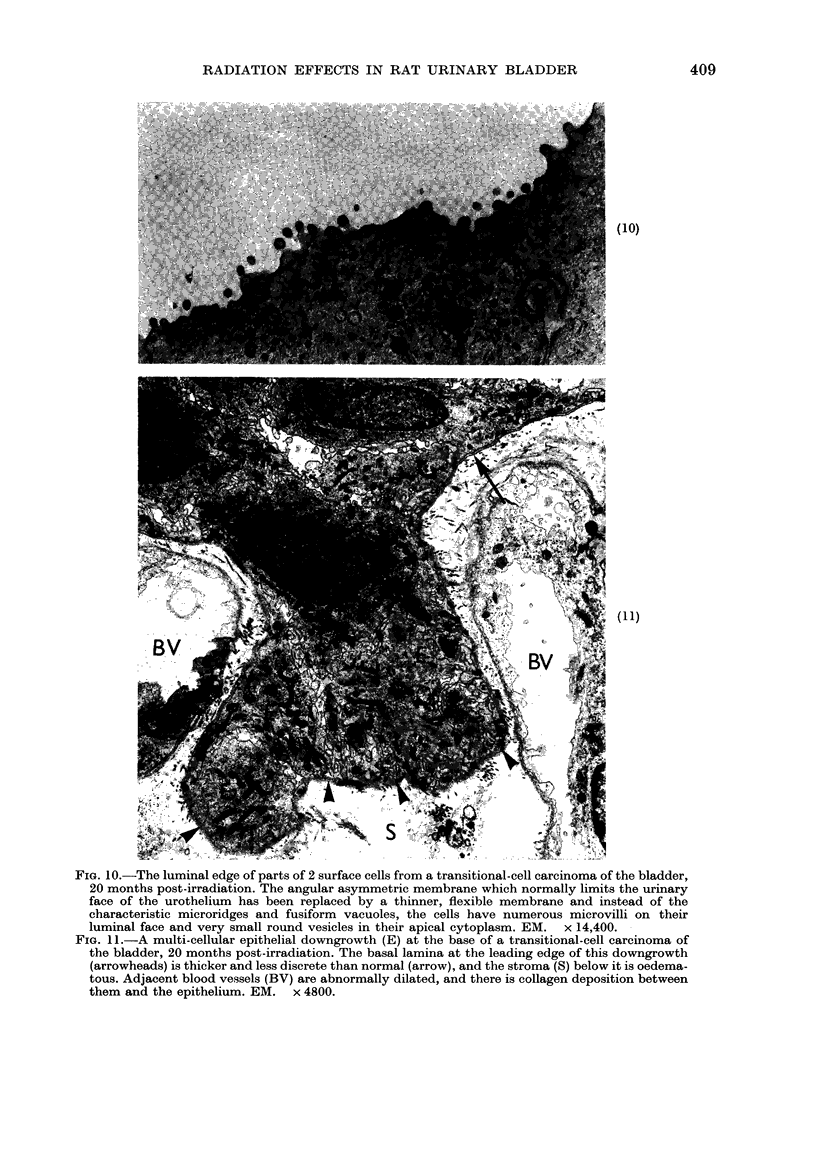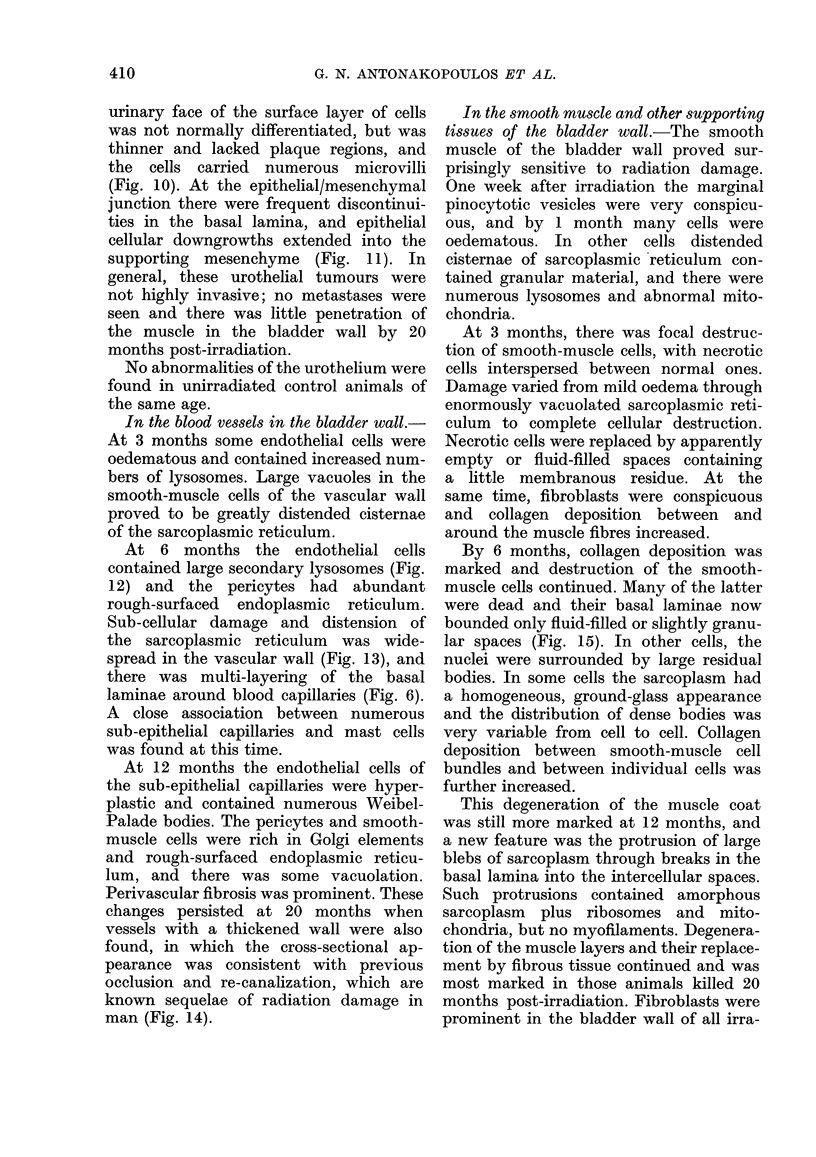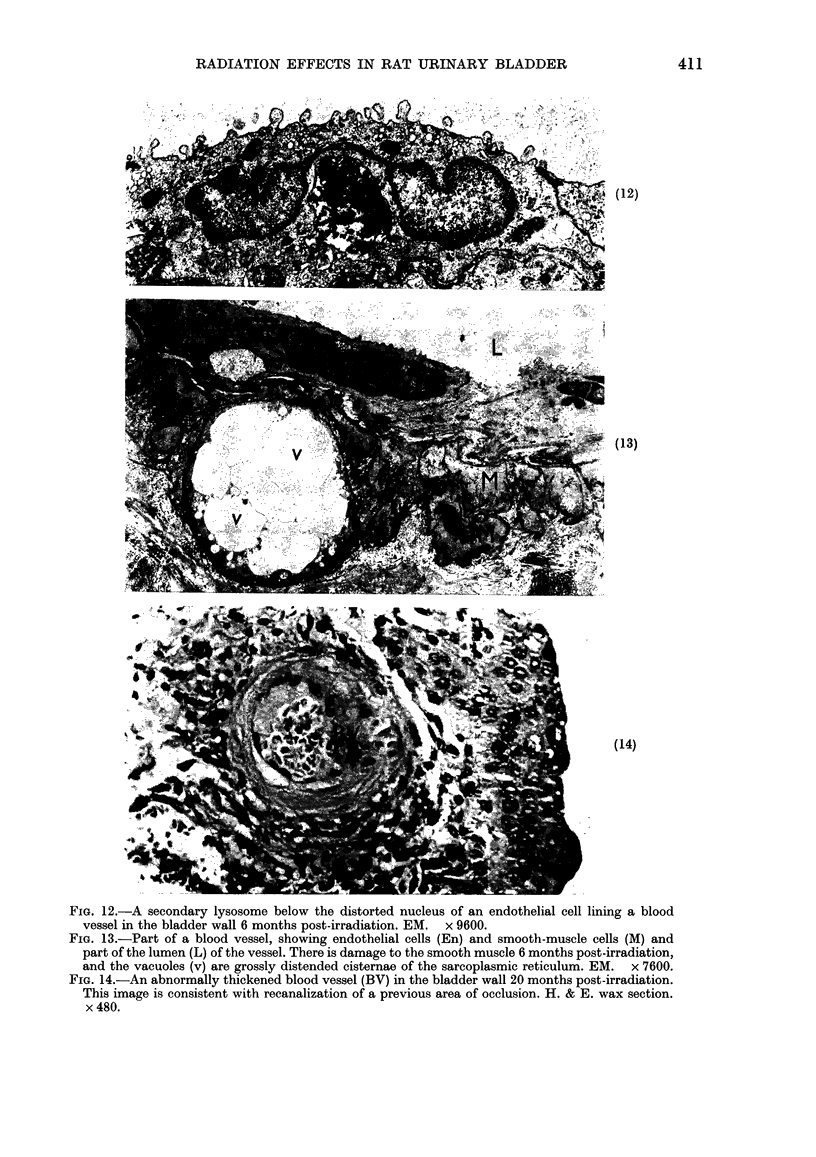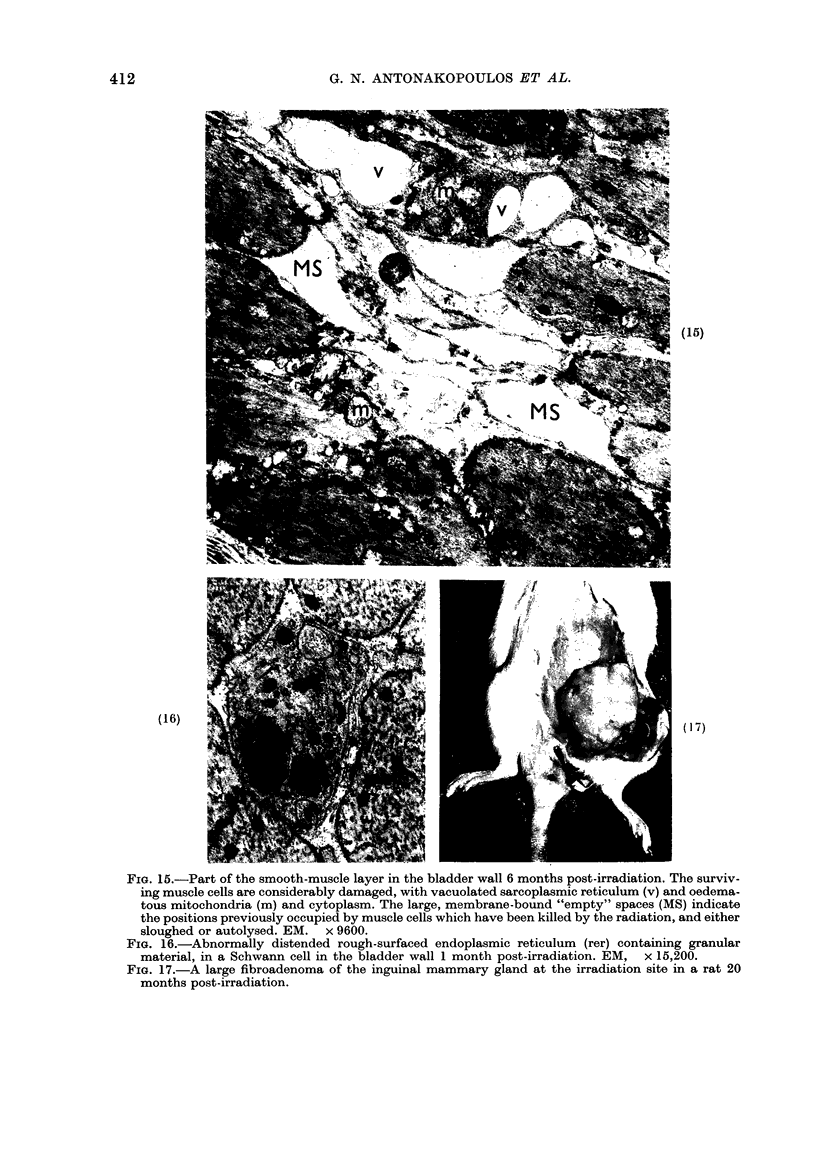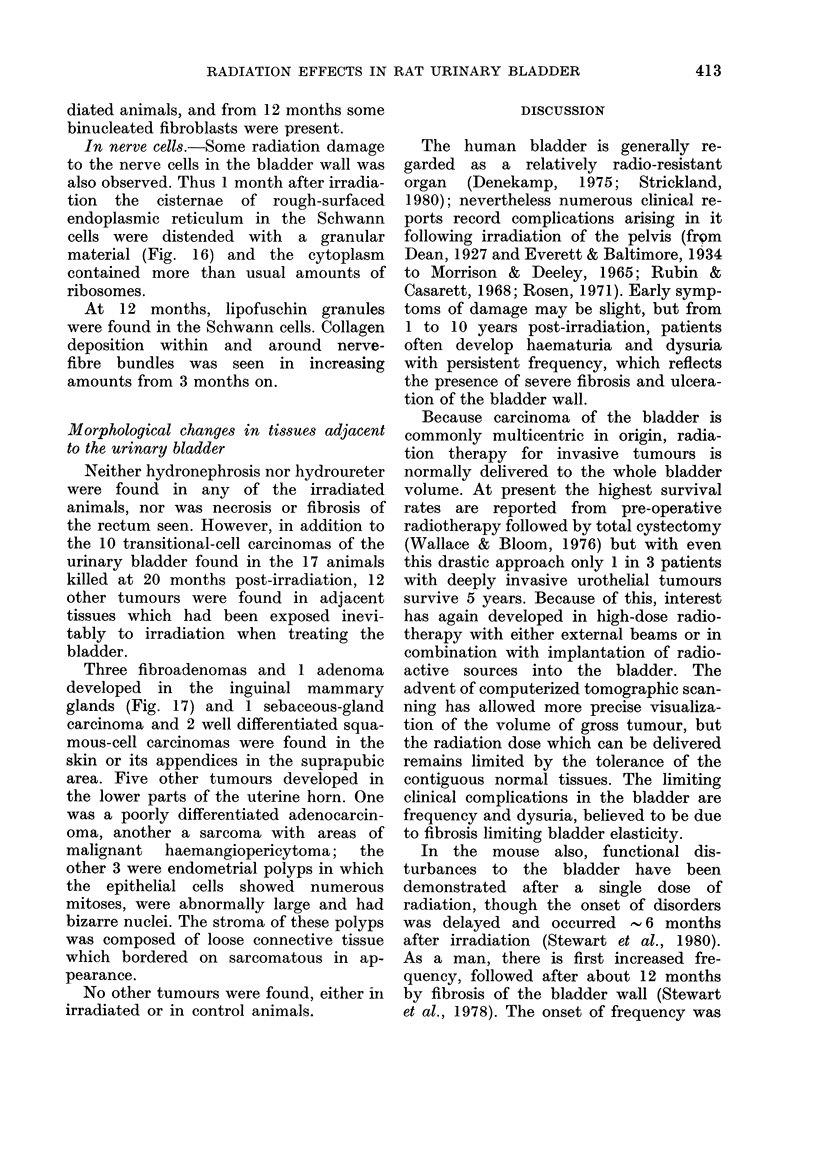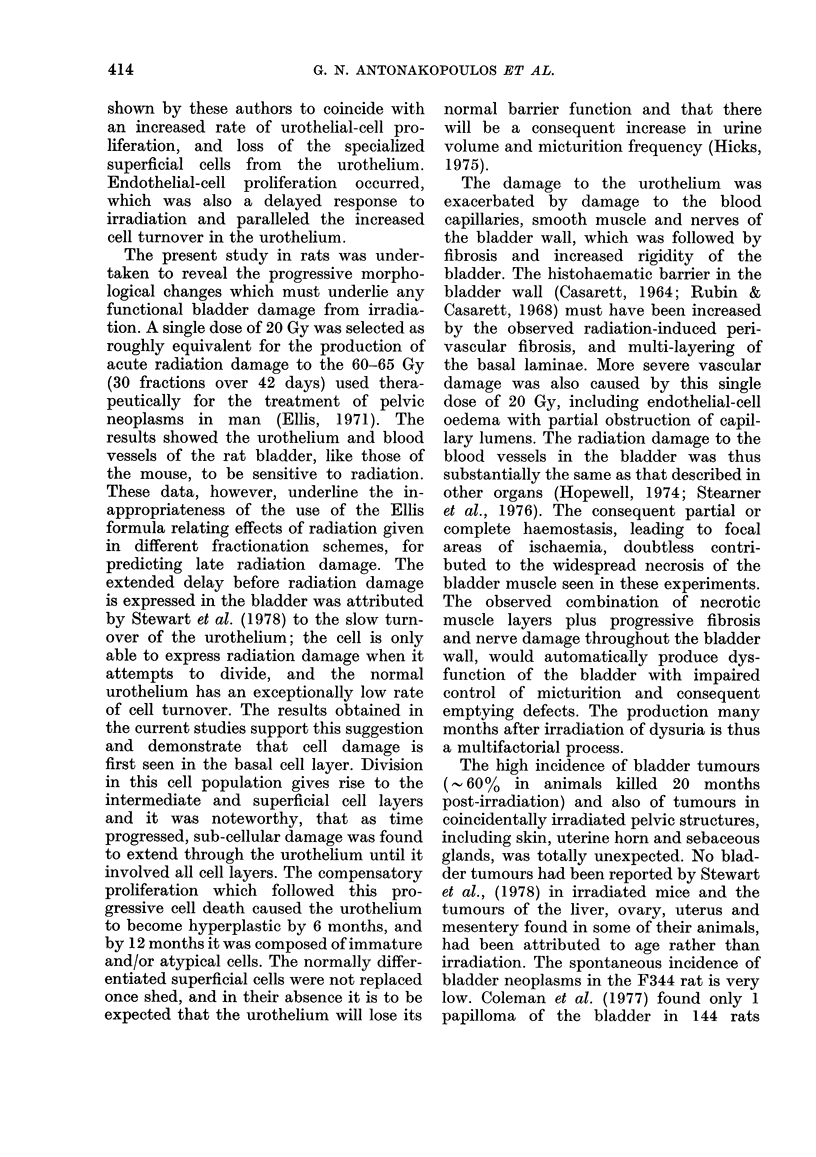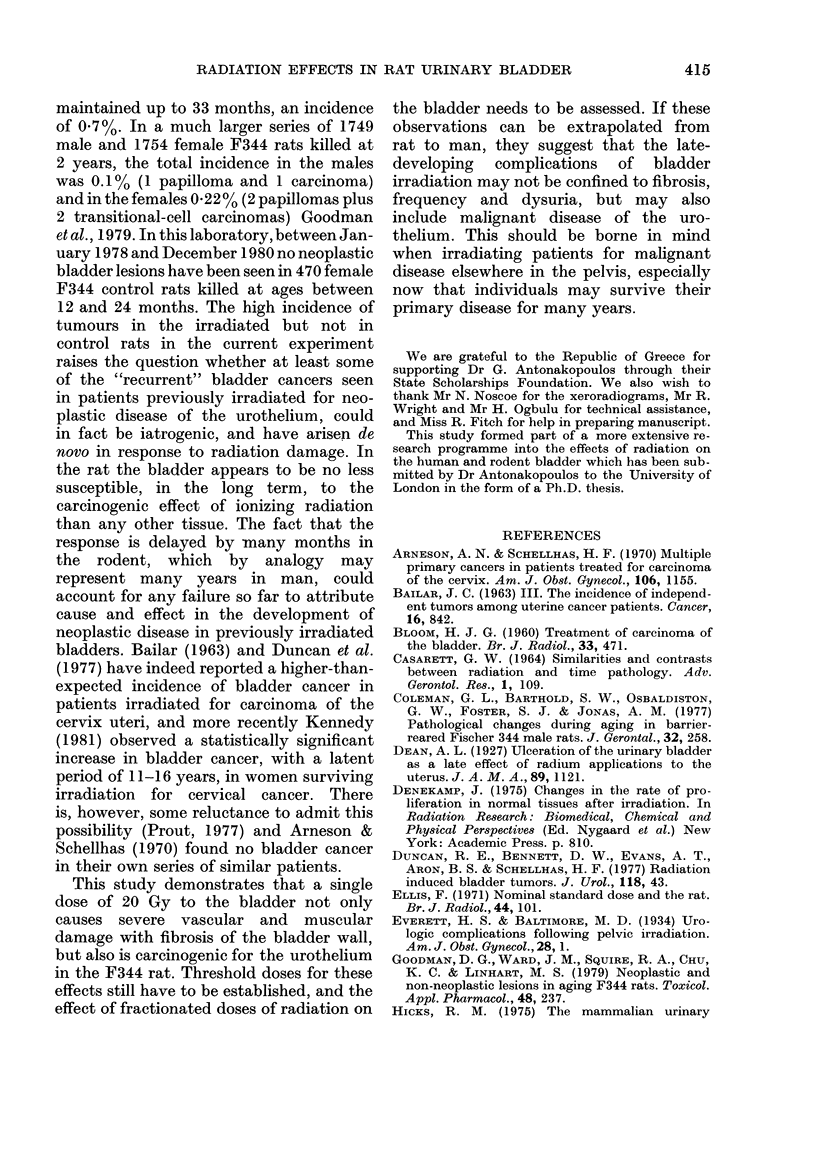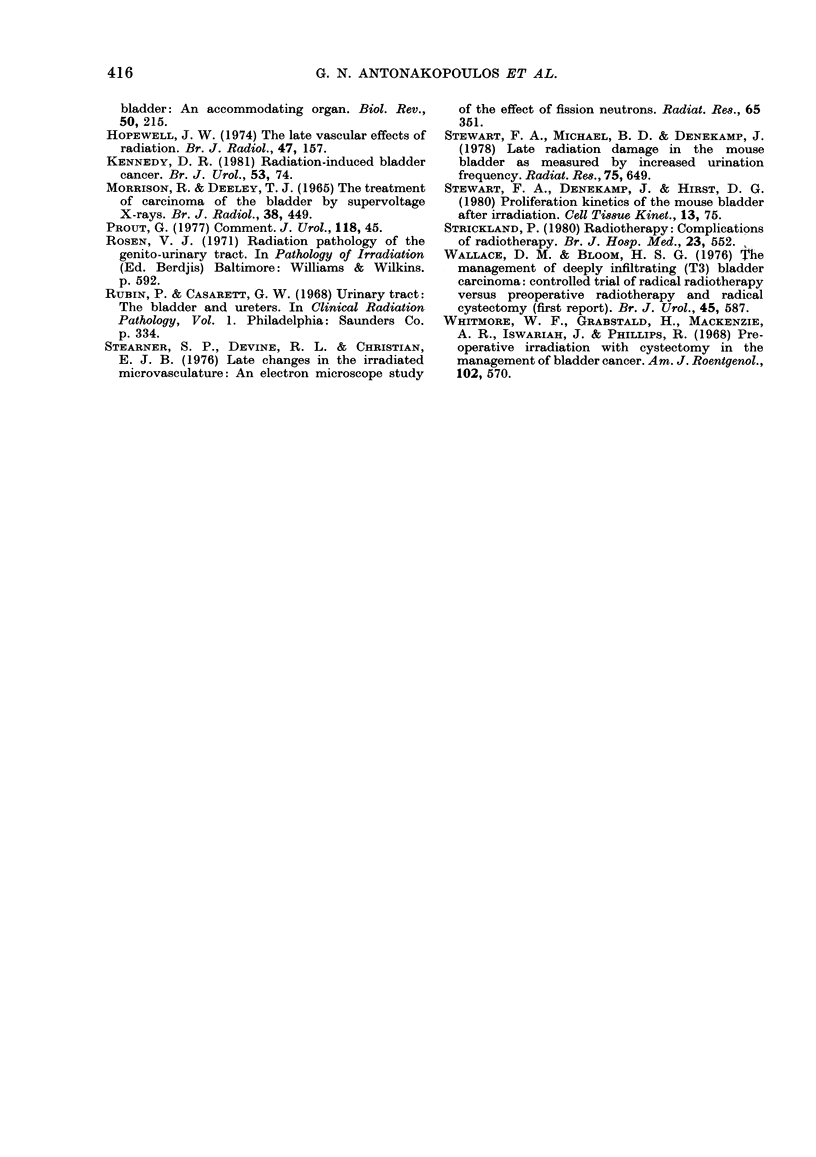# Early and late morphological changes (including carcinoma of the urothelium) induced by irradiation of the rat urinary bladder.

**DOI:** 10.1038/bjc.1982.217

**Published:** 1982-09

**Authors:** G. N. Antonakopoulos, R. M. Hicks, E. Hamilton, R. J. Berry

## Abstract

**Images:**


Br. J. Cancer (1982) 46, 403

### EARLY AND LATE MORPHOLOGICAL CHANGES (INCLUDING CARCINOMA OF THE UROTHELIUM) INDUCED BY IRRADIATION OF THE RAT URINARY BLADDER

G. N. ANTONAKOPOULOS*†, R. M. HICKS*, E. HAMILTON‡ AND R. J. BERRY‡

*From the *School of Pathology and ‡Department of Oncology, Middlesex Hospital Medical School, London W1P 7LD*

Received 25 January 1982 Accepted 29 April 1982

**Summary.—Effects of X-irradiating the urinary bladder of female F344 rats with a single dose of 20 Gy were studied by light and electron microscopy. The animals were killed 1 week–20 months post-irradiation, and all tissues of the bladder wall were found to be affected by the irradiation.**

**In the urothelium, damage was initially restricted to the basal cells but slowly extended to intermediate cells, and by 6 months post-irradiation the urothelium was focally hyperplastic. Twenty months post-irradiation, transitional-cell carcinomas were found in 10 of the surviving 17 animals (59%). The blood vessels in the bladder wall showed damage to both the endothelial cells and the smooth muscle. The fibroblasts in the connective tissue of the bladder wall appeared to show increased secretion after irradiation, and there was abundant collagen deposition, resulting in severe fibrosis of the bladder wall. After a latent period of a few months, focal degeneration and extensive necrosis of the smooth muscle cells were seen, leading to severe destruction and disorganization of the muscular coats of the bladder wall.**

**Thus, a single dose of irradiation of 20 Gy was sufficient to produce severe fibrosis of the bladder wall with smooth muscle degeneration and to induce carcinoma of the urothelium in most of the treated animals within 20 months.**

Carcinoma of the urothelium lining the bladder is the most frequent malignancy of the urinary tract in man, representing ~5% of all new malignancies in Western Europe and America. It is commoner in males and cigarette smokers. Usually presenting in middle life with painless haematuria, many bladder tumours are for long periods confined to the urothelium, with only superficial invasion into the musculature of the bladder wall. Although it is a multifocal disease with a high rate of recurrence, such superficial tumours may be treated successfully for some years through the cystoscope by surgical removal and/or fulguration. However, the disease commonly progresses both in stage and grade and once tumours penetrate more than half the thickness of the bladder wall, their ultimate prognosis becomes dismally poor. The only proven modes of treatment then are radiotherapy and/or total cystectomy (Bloom, 1960; Wallace & Bloom, 1976; Whitmore *et al.*, 1968).

The radical use of radiotherapy is limited by the tolerance of the whole bladder; even in patients whose tumours have been arrested, haematuria may recur in combination with dysuria and frequency, often many years after irradiation. The functional changes which develop in the bladder after irradiation have been studied in the mouse by Stewart *et al.* (1978) and some of the histological changes have been described. The present study was undertaken in the rat in order to explore the progressive

† Present address: Department of Histology, University of Athens Medical School, Athens, Greece.

morphological changes which underlie functional incapacity in the bladder due to irradiation.

### MATERIALS AND METHODS

*Method of irradiation.*—Female, 10-week-old F344 Fischer rats weighing ~175 g were used. It was necessary to irradiate the bladder without at the same time exposing the radiosensitive small intestine and rectum. In the rat, the urinary bladder is surrounded by thick folds of peritoneum, filled with adipose tissue. When distended, the bladder dome moves upwards and lies in close proximity to loops of the small intestine and caecum.

Animals were anaesthetized with i.p. pentobarbital ("Sagatal") at a dose of 30 mg/kg. Anaesthetized animals were laid on their side in a jig and the bowel massaged gently upwards. The hind legs were pulled as far back as possible in order to exclude the femur from the area to be irradiated. The jig, which was made from 2mm-thick lead sheet, immobilized the anaesthetized animal with its hind legs in the correct position, and also limited scattered radiation to the animal. The greater trochanter was located by palpation, and its position marked with ink on the overlying skin. The line joining the greater trochanter with the pubic symphysis was used as the lower edge of the radiation field. Although the trochanter is mobile, it lies close to the hip joint which is a stable point. Therefore the position of this line is constant and reliably situated. As is shown in a xeroradiogram (Fig. 1) when in the treatment position the entire urinary bladder lies above this line and hence was always included in the radiation field, even when the bladder was distended by excretion of the injected anaesthetic by the kidneys. After marking the exact site of the greater trochanter, animals were placed in pairs on a thick sheet of perspex. After covering the animal with a 5mm-thick lead shield to limit the X-ray beam, the mark over the greater trochanter was placed at the dorsal posterior corner of the window in this shield. The pubic symphysis was covered by the other posterior corner. The window was in the shape of a trapezium, with the dimensions shown in Fig. 2.

The bladder was irradiated with 250 kVp X-rays with 2mm aluminium added filtration at a dose rate of 1·975 Gy/min. Dose inhomogeneity through the bladder volume was less than $\pm 5\%$. After treatment animals were removed from the jig and returned to their cages. Care was taken to keep the animals warm to prevent hypothermia during anaesthesia. To check the accuracy of this method of locating the bladder, an anaesthetized animal was catheterized with 0·7mm plastic tubing (PP-10 tubing, Portex Ltd, Hythe, Kent) and injected under slight pressure with 0·25 ml of a radiopaque medium ("Conray 280" meglumine iothalamate). The greater trochanter was marked and the animal was covered by the lead shield without being put in the jig. A xeroradiogram was taken and, as is shown in Fig. 3, the entire bladder was included in the irradiated field.

*Histology and electron microscopy.*—Animals were killed at pre-selected times by cervical dislocation. The urinary bladder was exposed, emptied by gentle pressure and after clamping the urethra, cacodylate-buffered 4% formaldehyde (pH 7·3) was injected to fill, but not over distend, the bladder. The serosal surface of the bladder was bathed with the same fixative and after a few minutes the bladder was excised, opened and inspected for macroscopic abnormalities. Representative samples were further fixed in formalin for light microscopy or cut into ~1mm cubes and post-fixed in cold cacodylate-buffered 1% osmium tetroxide for electron microscopy. Adjacent organs, including loops of the caecum, small intestine and the uterus, were also processed for histology. All specimens for histology were fixed in formalin, embedded in paraffin wax, and sectioned and stained with haematoxylin and eosin. Thin sections (~80 nm) of Spurr-embedded bladder were contrast-stained with uranyl acetate and lead citrate for electron microscopy, and semi-thin (1 $\mu$m) sections were stained with toluidine blue for high-resolution light microscopy. For detailed examination of cellular structure thin sections were examined in a Jeol 100 electron microscope.

### RESULTS

### *Morphological changes in the urinary bladder*

*In the urothelium.*—The time-related changes in the urothelium are summarized

(1)

(2)

(3)

Fig. 1.—Xeroradiogram of unshielded rat to show position of the urinary bladder in relation to the greater trochanter (T) and the pubic symphysis (PS). The bladder was dilated with a radiopaque medium.

Fig. 2.—Dimensions of the trapezium-shaped window in the 2 mm-thick lead body shield, designed to reduce to a minimum the field of irradiation.

Fig. 3.—Xeroradiogram of rat, covered with the shield, to show the bladder included in the field of irradiation.

in the Table. One month post-irradiation, the urothelium appeared normal except for more-than-usual numbers of lysosomes in the basal layer. By 1 month, some basal cells were necrotic and macrophages had invaded the epithelium. The cell debris within the macrophages was probably of urothelial origin. A few binucleate basal cells were also seen at this time.

Three months post-irradiation, subcellular damage involved intermediate cells as well as basal cells. In both layers individual cells could be found containing very large lysosomes and associated areas of oedematous or rarified cytoplasm (Fig. 4) while others contained abundant smooth endoplasmic reticulum (Fig. 5) which is not conspicuous in normal urothelial cells. Binucleate basal cells were still present.

By 6 months, areas of focal hyperplasia were established but, in general, superficial cells were still normally differentiated, limited by the characteristic angular luminal cell membrane and contained fusiform vesicles in their apical cytoplasm. The deeper layers of smaller cells showed varying degrees of mild atypia, including nuclear irregularities, the presence of lipid droplets and varying amounts of endoplasmic reticulum, Golgi cisternae and mitochondria. The hemi-

TABLE.—*The effect of irradiation on the rat bladder urothelium*

| Time killed (mths) | Treatment | n | State of the urothelium: Normal | Hyperplastic | Neoplastic |
|---|---|---|---|---|---|
| ¼ | X-rays | 7 | 7 | | |
| | Control | 3 | 3 | | |
| 1 | X-rays | 7 | 7 | | |
| | Control | 3 | 3 | | |
| 3 | X-rays | 7 | 7 | | |
| | Control | 3 | 3 | | |
| 6 | X-rays | 7 | 4 | 3 | |
| | Control | 3 | 3 | | |
| 12 | X-rays | 7 | 3 | 4 | |
| | Control | 3 | 3 | | |
| 20* | X-rays | 17 | 3 | 4 | 10 |
| | Control | 3 | 3 | | |

* At this time, in addition to 10 transitional-cell carcinomas of the bladder, 12 other tumours were found, namely 3 fibroadenomas and an adenoma of the inguinal mammary gland; 1 sebaceous carcinoma and 2 squamous-cell carcinomas in the skin of the suprapubic area and its appendices, and 5 tumours of the uterus, including 1 adenocarcinoma, 1 sarcoma and 3 endometrial polyps.

desmosomes connecting the basal cells to the basal lamina were particularly conspicuous and appeared to have increased in number. The basal laminae were multi-layered (Fig. 6).

At 12 months, extensive atypical hyperplasia involved the urothelium in most animals (Fig. 7). The superficial cells at the surface of the epithelium were small, immature, and had lobulated nuclei, and most were no longer limited by the characteristic urothelial luminal membrane composed of plaques and hinge regions. Instead, they were limited by a thinner, flexible membrane, and many carried small microvilli on their luminal face. Fusiform cytoplasmic vacuoles were either totally absent, or very few. In the deeper cell layers there were increased numbers of ribosomes and cytoplasmic filaments and the latter were frequently aggregated into conspicuous tonofibrils. Mitochondria were elongated, and many contained deformed cristae whose axes were parallel to the long axis of the mitochondrion. There was hypertrophy of the Golgi complex, and lysosomes and residual bodies were more numerous than in controls. In the normal urothelium the epithelial/mesenchymal junction is relatively flat; in the bladder from animals irradiated 12 months before, this junction was folded, and large pseudopodia of the epithelium, still limited by a basal lamina, extended into the lamina propria. These down-growths were similar to those seen in tumours at 20 months (see below, Fig. 11).

At 20 months, all survivors were killed, and 10/17 had urothelial tumours, while 4 of the remaining 7 had multifocal, atypical hyperplasia. The tumours varied from simple, papillary outgrowths with a relatively well differentiated transitional cell structure (Fig. 8) to solid, invasive, less well differentiated carcinomas (Fig. 9). In most bladders there were multifocal tumours of the urothelium, and in all cases some at least were exophytic in growth pattern. Of the solid tumours, some had the growth pattern of inverted papillomas while others had a storiform growth pattern. Mitoses and nuclear pleomorphism were frequent. The subcellular features of the urothelial cells were comparable to those seen at 12 months, with abundant ribosomes, Golgi elements, mitochondria and cytoplasmic filaments either dispersed or organized into tonofibrils. The membrane on the

(4)

(5)

(6)

(7)

FIG. 4.—A damaged intermediate cell in the bladder urothelium, 3 months post-irradiation. Two large secondary lysosomes (arrows) are present in an otherwise oedematous cytoplasm. The few remaining sub-cellular organelles are displaced to the periphery of the cell. EM. ×3200.

FIG. 5.—Abundant smooth-surfaced endoplasmic reticulum (ser) in the cytoplasm of an intermediate cell, 3 months post-irradiation. EM. ×8000.

FIG. 6.—Section through part of a blood capillary at the base of the urothelium 6 months post-irradiation. The basal laminae (arrows) both around the capillary (C) and below the basal urothelial cells (B) are multilayered. EM. ×2560.

FIG. 7.—Part of the hyperplastic urothelium lining the bladder 12 months post-irradiation. The tissue shows an abnormal, differential growth pattern, with loss of normal cell differentiation and considerable nuclear pleomorphism. The junction between the urothelium and its supporting stroma is irregular, and sub-epithelial blood capillaries (C) and larger vessels (BV) are dilated and engorged. Toluidine blue-stained Epon section. ×192.

(8)

(9)

FIG. 8.—Section through one of the papillary fronds of a well-differentiated exophytic transitional-cell carcinoma of the bladder, found 20 months post-irradiation. Toluidine blue-stained Epon section. × 128.

FIG. 9.—Part of an invasive transitional-cell carcinoma of the bladder 20 months post-irradiation. The cords of epithelial cells growing into the stroma have a disorientated growth pattern, are less well differentiated than those covering the surface of the tumour and show some nuclear pleomorphism. Toluidine blue-stained Epon section. × 112.

(10)

(11)

FIG. 10.—The luminal edge of parts of 2 surface cells from a transitional-cell carcinoma of the bladder, 20 months post-irradiation. The angular asymmetric membrane which normally limits the urinary face of the urothelium has been replaced by a thinner, flexible membrane and instead of the characteristic microridges and fusiform vacuoles, the cells have numerous microvilli on their luminal face and very small round vesicles in their apical cytoplasm. EM. × 14,400.

FIG. 11.—A multi-cellular epithelial downgrowth (E) at the base of a transitional-cell carcinoma of the bladder, 20 months post-irradiation. The basal lamina at the leading edge of this downgrowth (arrowheads) is thicker and less discrete than normal (arrow), and the stroma (S) below it is oedematous. Adjacent blood vessels (BV) are abnormally dilated, and there is collagen deposition between them and the epithelium. EM. × 4800.

urinary face of the surface layer of cells was not normally differentiated, but was thinner and lacked plaque regions, and the cells carried numerous microvilli (Fig. 10). At the epithelial/mesenchymal junction there were frequent discontinuities in the basal lamina, and epithelial cellular downgrowths extended into the supporting mesenchyme (Fig. 11). In general, these urothelial tumours were not highly invasive; no metastases were seen and there was little penetration of the muscle in the bladder wall by 20 months post-irradiation.

No abnormalities of the urothelium were found in unirradiated control animals of the same age.

*In the blood vessels in the bladder wall.*—At 3 months some endothelial cells were oedematous and contained increased numbers of lysosomes. Large vacuoles in the smooth-muscle cells of the vascular wall proved to be greatly distended cisternae of the sarcoplasmic reticulum.

At 6 months the endothelial cells contained large secondary lysosomes (Fig. 12) and the pericytes had abundant rough-surfaced endoplasmic reticulum. Sub-cellular damage and distension of the sarcoplasmic reticulum was widespread in the vascular wall (Fig. 13), and there was multi-layering of the basal laminae around blood capillaries (Fig. 6). A close association between numerous sub-epithelial capillaries and mast cells was found at this time.

At 12 months the endothelial cells of the sub-epithelial capillaries were hyperplastic and contained numerous Weibel-Palade bodies. The pericytes and smooth-muscle cells were rich in Golgi elements and rough-surfaced endoplasmic reticulum, and there was some vacuolation. Perivascular fibrosis was prominent. These changes persisted at 20 months when vessels with a thickened wall were also found, in which the cross-sectional appearance was consistent with previous occlusion and re-canalization, which are known sequelae of radiation damage in man (Fig. 14).

*In the smooth muscle and other supporting tissues of the bladder wall.*—The smooth muscle of the bladder wall proved surprisingly sensitive to radiation damage. One week after irradiation the marginal pinocytotic vesicles were very conspicuous, and by 1 month many cells were oedematous. In other cells distended cisternae of sarcoplasmic reticulum contained granular material, and there were numerous lysosomes and abnormal mitochondria.

At 3 months, there was focal destruction of smooth-muscle cells, with necrotic cells interspersed between normal ones. Damage varied from mild oedema through enormously vacuolated sarcoplasmic reticulum to complete cellular destruction. Necrotic cells were replaced by apparently empty or fluid-filled spaces containing a little membranous residue. At the same time, fibroblasts were conspicuous and collagen deposition between and around the muscle fibres increased.

By 6 months, collagen deposition was marked and destruction of the smooth-muscle cells continued. Many of the latter were dead and their basal laminae now bounded only fluid-filled or slightly granular spaces (Fig. 15). In other cells, the nuclei were surrounded by large residual bodies. In some cells the sarcoplasm had a homogeneous, ground-glass appearance and the distribution of dense bodies was very variable from cell to cell. Collagen deposition between smooth-muscle cell bundles and between individual cells was further increased.

This degeneration of the muscle coat was still more marked at 12 months, and a new feature was the protrusion of large blebs of sarcoplasm through breaks in the basal lamina into the intercellular spaces. Such protrusions contained amorphous sarcoplasm plus ribosomes and mitochondria, but no myofilaments. Degeneration of the muscle layers and their replacement by fibrous tissue continued and was most marked in those animals killed 20 months post-irradiation. Fibroblasts were prominent in the bladder wall of all irra-

(12)

(13)

(14)

FIG. 12.—A secondary lysosome below the distorted nucleus of an endothelial cell lining a blood vessel in the bladder wall 6 months post-irradiation. EM. × 9600.

FIG. 13.—Part of a blood vessel, showing endothelial cells (En) and smooth-muscle cells (M) and part of the lumen (L) of the vessel. There is damage to the smooth muscle 6 months post-irradiation, and the vacuoles (v) are grossly distended cisternae of the sarcoplasmic reticulum. EM. × 7600.

FIG. 14.—An abnormally thickened blood vessel (BV) in the bladder wall 20 months post-irradiation. This image is consistent with recanalization of a previous area of occlusion. H. & E. wax section. × 480.

(15)

(16)

(17)

FIG. 15.—Part of the smooth-muscle layer in the bladder wall 6 months post-irradiation. The surviving muscle cells are considerably damaged, with vacuolated sarcoplasmic reticulum (v) and oedematous mitochondria (m) and cytoplasm. The large, membrane-bound "empty" spaces (MS) indicate the positions previously occupied by muscle cells which have been killed by the radiation, and either sloughed or autolysed. EM. × 9600.

FIG. 16.—Abnormally distended rough-surfaced endoplasmic reticulum (rer) containing granular material, in a Schwann cell in the bladder wall 1 month post-irradiation. EM, × 15,200.

FIG. 17.—A large fibroadenoma of the inguinal mammary gland at the irradiation site in a rat 20 months post-irradiation.

diated animals, and from 12 months some binucleated fibroblasts were present.

*In nerve cells.*—Some radiation damage to the nerve cells in the bladder wall was also observed. Thus 1 month after irradiation the cisternae of rough-surfaced endoplasmic reticulum in the Schwann cells were distended with a granular material (Fig. 16) and the cytoplasm contained more than usual amounts of ribosomes.

At 12 months, lipofuschin granules were found in the Schwann cells. Collagen deposition within and around nerve-fibre bundles was seen in increasing amounts from 3 months on.

*Morphological changes in tissues adjacent to the urinary bladder*

Neither hydronephrosis nor hydroureter were found in any of the irradiated animals, nor was necrosis or fibrosis of the rectum seen. However, in addition to the 10 transitional-cell carcinomas of the urinary bladder found in the 17 animals killed at 20 months post-irradiation, 12 other tumours were found in adjacent tissues which had been exposed inevitably to irradiation when treating the bladder.

Three fibroadenomas and 1 adenoma developed in the inguinal mammary glands (Fig. 17) and 1 sebaceous-gland carcinoma and 2 well differentiated squamous-cell carcinomas were found in the skin or its appendices in the suprapubic area. Five other tumours developed in the lower parts of the uterine horn. One was a poorly differentiated adenocarcinoma, another a sarcoma with areas of malignant haemangiopericytoma; the other 3 were endometrial polyps in which the epithelial cells showed numerous mitoses, were abnormally large and had bizarre nuclei. The stroma of these polyps was composed of loose connective tissue which bordered on sarcomatous in appearance.

No other tumours were found, either in irradiated or in control animals.

### DISCUSSION

The human bladder is generally regarded as a relatively radio-resistant organ (Denekamp, 1975; Strickland, 1980); nevertheless numerous clinical reports record complications arising in it following irradiation of the pelvis (from Dean, 1927 and Everett & Baltimore, 1934 to Morrison & Deeley, 1965; Rubin & Casarett, 1968; Rosen, 1971). Early symptoms of damage may be slight, but from 1 to 10 years post-irradiation, patients often develop haematuria and dysuria with persistent frequency, which reflects the presence of severe fibrosis and ulceration of the bladder wall.

Because carcinoma of the bladder is commonly multicentric in origin, radiation therapy for invasive tumours is normally delivered to the whole bladder volume. At present the highest survival rates are reported from pre-operative radiotherapy followed by total cystectomy (Wallace & Bloom, 1976) but with even this drastic approach only 1 in 3 patients with deeply invasive urothelial tumours survive 5 years. Because of this, interest has again developed in high-dose radiotherapy with either external beams or in combination with implantation of radioactive sources into the bladder. The advent of computerized tomographic scanning has allowed more precise visualization of the volume of gross tumour, but the radiation dose which can be delivered remains limited by the tolerance of the contiguous normal tissues. The limiting clinical complications in the bladder are frequency and dysuria, believed to be due to fibrosis limiting bladder elasticity.

In the mouse also, functional disturbances to the bladder have been demonstrated after a single dose of radiation, though the onset of disorders was delayed and occurred ~6 months after irradiation (Stewart *et al.*, 1980). As a man, there is first increased frequency, followed after about 12 months by fibrosis of the bladder wall (Stewart *et al.*, 1978). The onset of frequency was shown by these authors to coincide with an increased rate of urothelial-cell proliferation, and loss of the specialized superficial cells from the urothelium. Endothelial-cell proliferation occurred, which was also a delayed response to irradiation and paralleled the increased cell turnover in the urothelium.

The present study in rats was undertaken to reveal the progressive morphological changes which must underlie any functional bladder damage from irradiation. A single dose of 20 Gy was selected as roughly equivalent for the production of acute radiation damage to the 60–65 Gy (30 fractions over 42 days) used therapeutically for the treatment of pelvic neoplasms in man (Ellis, 1971). The results showed the urothelium and blood vessels of the rat bladder, like those of the mouse, to be sensitive to radiation. These data, however, underline the inappropriateness of the use of the Ellis formula relating effects of radiation given in different fractionation schemes, for predicting late radiation damage. The extended delay before radiation damage is expressed in the bladder was attributed by Stewart *et al.* (1978) to the slow turnover of the urothelium; the cell is only able to express radiation damage when it attempts to divide, and the normal urothelium has an exceptionally low rate of cell turnover. The results obtained in the current studies support this suggestion and demonstrate that cell damage is first seen in the basal cell layer. Division in this cell population gives rise to the intermediate and superficial cell layers and it was noteworthy, that as time progressed, sub-cellular damage was found to extend through the urothelium until it involved all cell layers. The compensatory proliferation which followed this progressive cell death caused the urothelium to become hyperplastic by 6 months, and by 12 months it was composed of immature and/or atypical cells. The normally differentiated superficial cells were not replaced once shed, and in their absence it is to be expected that the urothelium will lose its normal barrier function and that there will be a consequent increase in urine volume and micturition frequency (Hicks, 1975).

The damage to the urothelium was exacerbated by damage to the blood capillaries, smooth muscle and nerves of the bladder wall, which was followed by fibrosis and increased rigidity of the bladder. The histohaematic barrier in the bladder wall (Casarett, 1964; Rubin & Casarett, 1968) must have been increased by the observed radiation-induced perivascular fibrosis, and multi-layering of the basal laminae. More severe vascular damage was also caused by this single dose of 20 Gy, including endothelial-cell oedema with partial obstruction of capillary lumens. The radiation damage to the blood vessels in the bladder was thus substantially the same as that described in other organs (Hopewell, 1974; Stearner *et al.*, 1976). The consequent partial or complete haemostasis, leading to focal areas of ischaemia, doubtless contributed to the widespread necrosis of the bladder muscle seen in these experiments. The observed combination of necrotic muscle layers plus progressive fibrosis and nerve damage throughout the bladder wall, would automatically produce dysfunction of the bladder with impaired control of micturition and consequent emptying defects. The production many months after irradiation of dysuria is thus a multifactorial process.

The high incidence of bladder tumours ($\sim 60\%$ in animals killed 20 months post-irradiation) and also of tumours in coincidentally irradiated pelvic structures, including skin, uterine horn and sebaceous glands, was totally unexpected. No bladder tumours had been reported by Stewart *et al.*, (1978) in irradiated mice and the tumours of the liver, ovary, uterus and mesentery found in some of their animals, had been attributed to age rather than irradiation. The spontaneous incidence of bladder neoplasms in the F344 rat is very low. Coleman *et al.* (1977) found only 1 papilloma of the bladder in 144 rats maintained up to 33 months, an incidence of 0·7%. In a much larger series of 1749 male and 1754 female F344 rats killed at 2 years, the total incidence in the males was 0.1% (1 papilloma and 1 carcinoma) and in the females 0·22% (2 papillomas plus 2 transitional-cell carcinomas) Goodman *et al.*, 1979. In this laboratory, between January 1978 and December 1980 no neoplastic bladder lesions have been seen in 470 female F344 control rats killed at ages between 12 and 24 months. The high incidence of tumours in the irradiated but not in control rats in the current experiment raises the question whether at least some of the "recurrent" bladder cancers seen in patients previously irradiated for neoplastic disease of the urothelium, could in fact be iatrogenic, and have arisen *de novo* in response to radiation damage. In the rat the bladder appears to be no less susceptible, in the long term, to the carcinogenic effect of ionizing radiation than any other tissue. The fact that the response is delayed by many months in the rodent, which by analogy may represent many years in man, could account for any failure so far to attribute cause and effect in the development of neoplastic disease in previously irradiated bladders. Bailar (1963) and Duncan *et al.* (1977) have indeed reported a higher-than-expected incidence of bladder cancer in patients irradiated for carcinoma of the cervix uteri, and more recently Kennedy (1981) observed a statistically significant increase in bladder cancer, with a latent period of 11–16 years, in women surviving irradiation for cervical cancer. There is, however, some reluctance to admit this possibility (Prout, 1977) and Arneson & Schellhas (1970) found no bladder cancer in their own series of similar patients.

This study demonstrates that a single dose of 20 Gy to the bladder not only causes severe vascular and muscular damage with fibrosis of the bladder wall, but also is carcinogenic for the urothelium in the F344 rat. Threshold doses for these effects still have to be established, and the effect of fractionated doses of radiation on the bladder needs to be assessed. If these observations can be extrapolated from rat to man, they suggest that the late-developing complications of bladder irradiation may not be confined to fibrosis, frequency and dysuria, but may also include malignant disease of the urothelium. This should be borne in mind when irradiating patients for malignant disease elsewhere in the pelvis, especially now that individuals may survive their primary disease for many years.

We are grateful to the Republic of Greece for supporting Dr G. Antonakopoulos through their State Scholarships Foundation. We also wish to thank Mr N. Noscoe for the xeroradiograms, Mr R. Wright and Mr H. Ogbulu for technical assistance, and Miss R. Fitch for help in preparing manuscript.

This study formed part of a more extensive research programme into the effects of radiation on the human and rodent bladder which has been submitted by Dr Antonakopoulos to the University of London in the form of a Ph.D. thesis.

### REFERENCES

ARNESON, A. N. & SCHELLHAS, H. F. (1970) Multiple primary cancers in patients treated for carcinoma of the cervix. *Am. J. Obst. Gynecol.*, **106**, 1155.

BAILAR, J. C. (1963) III. The incidence of independent tumors among uterine cancer patients. *Cancer*, **16**, 842.

BLOOM, H. J. G. (1960) Treatment of carcinoma of the bladder. *Br. J. Radiol.*, **33**, 471.

CASARETT, G. W. (1964) Similarities and contrasts between radiation and time pathology. *Adv. Gerontol. Res.*, **1**, 109.

COLEMAN, G. L., BARTHOLD, S. W., OSBALDISTON, G. W., FOSTER, S. J. & JONAS, A. M. (1977) Pathological changes during aging in barrier-reared Fischer 344 male rats. *J. Gerontal.*, **32**, 258.

DEAN, A. L. (1927) Ulceration of the urinary bladder as a late effect of radium applications to the uterus. *J. A. M. A.*, **89**, 1121.

DENEKAMP, J. (1975) Changes in the rate of proliferation in normal tissues after irradiation. In *Radiation Research: Biomedical, Chemical and Physical Perspectives* (Ed. Nygaard *et al.*) New York: Academic Press. p. 810.

DUNCAN, R. E., BENNETT, D. W., EVANS, A. T., ARON, B. S. & SCHELLHAS, H. F. (1977) Radiation induced bladder tumors. *J. Urol.*, **118**, 43.

ELLIS, F. (1971) Nominal standard dose and the rat. *Br. J. Radiol.*, **44**, 101.

EVERETT, H. S. & BALTIMORE, M. D. (1934) Urologic complications following pelvic irradiation. *Am. J. Obst. Gynecol.*, **28**, 1.

GOODMAN, D. G., WARD, J. M., SQUIRE, R. A., CHU, K. C. & LINHART, M. S. (1979) Neoplastic and non-neoplastic lesions in aging F344 rats. *Toxicol. Appl. Pharmacol.*, **48**, 237.

HICKS, R. M. (1975) The mammalian urinary bladder: An accommodating organ. *Biol. Rev.*, **50,** 215.

HOPEWELL, J. W. (1974) The late vascular effects of radiation. *Br. J. Radiol.*, **47,** 157.

KENNEDY, D. R. (1981) Radiation-induced bladder cancer. *Br. J. Urol.*, **53,** 74.

MORRISON, R. & DEELEY, T. J. (1965) The treatment of carcinoma of the bladder by supervoltage X-rays. *Br. J. Radiol.*, **38,** 449.

PROUT, G. (1977) Comment. *J. Urol.*, **118,** 45.

ROSEN, V. J. (1971) Radiation pathology of the genito-urinary tract. In *Pathology of Irradiation* (Ed. Berdjis) Baltimore: Williams & Wilkins. p. 592.

RUBIN, P. & CASARETT, G. W. (1968) Urinary tract: The bladder and ureters. In *Clinical Radiation Pathology, Vol.* 1. Philadelphia: Saunders Co. p. 334.

STEARNER, S. P., DEVINE, R. L. & CHRISTIAN, E. J. B. (1976) Late changes in the irradiated microvasculature: An electron microscope study of the effect of fission neutrons. *Radiat. Res.*, **65** 351.

STEWART, F. A., MICHAEL, B. D. & DENEKAMP, J. (1978) Late radiation damage in the mouse bladder as measured by increased urination frequency. *Radiat. Res.*, **75,** 649.

STEWART, F. A., DENEKAMP, J. & HIRST, D. G. (1980) Proliferation kinetics of the mouse bladder after irradiation. *Cell Tissue Kinet.*, **13,** 75.

STRICKLAND, P. (1980) Radiotherapy: Complications of radiotherapy. *Br. J. Hosp. Med.*, **23,** 552.

WALLACE, D. M. & BLOOM, H. S. G. (1976) The management of deeply infiltrating (T3) bladder carcinoma: controlled trial of radical radiotherapy versus preoperative radiotherapy and radical cystectomy (first report). *Br. J. Urol.*, **45,** 587.

WHITMORE, W. F., GRABSTALD, H., MACKENZIE, A. R., ISWARIAH, J. & PHILLIPS, R. (1968) Preoperative irradiation with cystectomy in the management of bladder cancer. *Am. J. Roentgenol.*, **102,** 570.